# mTOR inhibitors effects on regulatory T cells and on dendritic cells

**DOI:** 10.1186/s12967-016-0916-7

**Published:** 2016-05-31

**Authors:** Giovanni Stallone, Barbara Infante, Adelaide Di Lorenzo, Federica Rascio, Gianluigi Zaza, Giuseppe Grandaliano

**Affiliations:** Nephrology, Dialysis and Tranplantation Unit, Department of Medical and Surgical Sciences, University of Foggia, Viale Luigi Pinto, 1, 71100 Foggia, Italy; Renal Unit, Department of Medicine, University-Hospital of Verona, University of Verona, Piazzale A. Stefani 1, 37126 Verona, VR Italy

**Keywords:** mTOR inhibitors, Treg, Dendritic cells, Operational tolerance, Transplantation

## Abstract

The mammalian target of rapamycin (mTOR), a cytoplasmic serine/threonine kinase, represents a key biologic “switch” modulating cell metabolisms in response to environmental signals and is now recognized as a central regulator of the immune system. There is an increasing body of evidence supporting the hypothesis that mTOR inhibitors exhibit several biological properties in addition to immunosuppression, including anti-neoplastic effects, cardio-protective activities, and an array of immunomodulatory actions facilitating the development of an operational graft tolerance. The biological mechanisms explaining how mTOR inhibition can enable a tolerogenic state are still largely unclear. The induction of transplant tolerance might at the same time decrease rejection rate and minimize immunosuppression-related side effects, leading to an improvement in long-term graft outcome. In this scenario, T cell immunoregulation has been defined as the hallmark of peripheral tolerance. Two main immunologic cell populations have been reported to play a central role in this setting: regulatory T cells (Tregs) and dendritic cells (DCs). In this review we focus on mTOR inhibitors effects on *Treg and DCs* differentiation, activation, and function in the transplantation setting.

## Background

Allograft rejection is a complex array of events involving both cellular and humoral adaptive immune response. This process is primed by the recognition of non-self histocompatibility antigens expressed within the graft. Recipient T cells may recognize graft major histocompatibility complex (MHC) antigen through two main routes. The direct pathway is characterized by the presentation of alloantigen to recipient T cells by donor antigen-presenting cells (APC). The indirect pathway involves the presentation of MHC alloantigen to T cells by recipient APCs [1–3]. Rejection is triggered by the clonal expansion and activation of a T cell population recognizing one or more immunodominant peptides derived from the hypervariable region of donor’s HLA molecules. T cells, that recognize epitopes of the same molecule or of another HLA antigen, become activated and contribute to the amplification and maintenance of the allograft specific immune reaction [1–3].

Although leukocyte populations, including B cells and T cells, dendritic cells (DCs) and endothelial cells (ECs) can contribute to the destruction of the graft, they can also promote a tolerogenic immune response supporting long-term graft survival [3]. Regulatory cells that prevent allograft rejection are specialized leukocyte populations, selected during their development to present regulatory functions, and acquiring a tolerogenic phenotype in the graft microenvironment or in the graft-draining lymph nodes [3]. Operational transplantation tolerance has been defined as a stable graft function without signs of rejection in the presence of a minimal immunosuppression [4].

The mammalian target of rapamycin (mTOR), a cytoplasmic serine/threonine kinase, represents a key biologic “switch” modulating cell metabolisms in response to environmental signals and is now recognized as a central regulator of the immune system. Indeed, mTOR is a central node in the signaling pathways integrating several environmental cues in the immune microenvironment. Rapamycin or Sirolimus, the first mTOR inhibitor, born as an antibiotic, was found to present powerful immunosuppressive effects. Rapamycin’s effects on mTOR depend on its ability to bind to the immunophilin FKBP12 [5], the same bound by the calcineurin inhibitor (CNI) tacrolimus. However, unlike tacrolimus, rapamycin does not influence calcineurin activity and, thus, does not interfere with T cell receptor-induced NF-AT nuclear translocation. Originally, the immunosuppressive properties of rapamycin were believed to be due to its ability to inhibit T cell proliferation. Indeed, mTOR activation induces the degradation of the cell cycle inhibitor p27 along with an increase in cyclin D3 expression [5, 6]. Several experimental models suggested that T cell anergy was the result of T cell receptor (TCR) engagement, in the absence of proliferation and that IL-2-induced cell proliferation could induced a reversal of T cell anergy [7]. Since rapamycin inhibits cell proliferation, it was hypothesized that mTOR inhibitors’ immunosuppressive effects might be, at least partially, due to their ability to promote anergy. Rapamycin can, indeed, induce T cell anergy even in the presence of a valid co-stimulation signal [8]. Interestingly, concomitant inhibition of calcineurin blocked rapamycin-induced anergy [8, 9]. This observation highlights the fact that although CNI are potent suppressors of T cell activation, they may also prevent T cell tolerance [10]. However, the inhibition of cell proliferation is not the only mechanism underlying rapamycin-induced anergy. Indeed, cell cycle arrest in G1 in the absence of mTOR inhibition did not induce anergy, while inducing T cell proliferation in the presence of rapamycin was unable to overcome anergy [8, 9]. On the basis of these observations it is conceivable that the modulation of several signals controlled by mTOR may influence the activity of the two main cell types involved in transplantation tolerance, regulatory T cells (Tregs) and DCs.

### Tregs and mTOR inhibition

Different T cell populations with regulatory activity might contribute to prevention of allograft rejection, including naïve T cells, CD4^+^ T cells, CD8^+^ T cells, CD4^−^ CD8^–^ T cells [11]. Naïve T cells require antigen presentation and co-stimulation for complete activation. When TCR bind the antigen within the MHC in the absence of co-stimulation, T cells neither proliferate nor produce IL-2. In addition, they remain unresponsive on rechallenge with the same antigen becoming anergic [7].

CD4^+^CD25^+^ Tregs are constitutively present in every healthy subject and express high levels of forkhead box P3 (FOXP3), a key transcription factor for the development and function of these cells establishing a pattern of Tregs-specific gene expression [12, 13]. In solid-organ transplant recipients, there is an inadequate number of Tregs to prevent allograft rejection, in particular when donor-specific memory T cells specific are present. The high frequency of alloantigen-reactive T cells compared with the relatively small number of Treg cells promote an unbalance in the immune repertoire of the recipient leading to allograft destruction. The balance between graft rejection and regulation can be modulated, either before or after transplantation, using strategies based on the inhibition of effector T cells and/or on the increase of frequency or functional activity of alloantigen-reactive Tregs [14]. Tregs express on their cell surface high levels of CD25, CD122 and 132, the alpha, beta and gamma chain, respectively, of IL-2 [15]. Although Tregs do not secrete this cytokine, this high, constitutive IL-2 receptor expression suggests a great sensitivity of Tregs to this cytokine for both their activation and survival. In fact, Tregs may consume IL-2 in the microenvironment reducing the availability of this cytokine to effector T cells, thus causing their apoptosis [15]. On the other hand, Tregs immunosuppressive effects were associated with their secretion of suppressive cytokines or receptors, including transforming growth factor-beta (TGF-β), IL-10, IL-35 and cytotoxic T lymphocyte antigen 4 (CTLA-4). Indeed, IL-10 or CTLA-4 inhibition results in a significantly decreased protection of the graft by Tregs, suggesting that IL-10 and CTLA-4 represent key mediators for the regulatory effects of this cell type [15].

There are several types of human CD8^+^ suppressor T cells, mainly (i) CD8^+^ CD28^−^ FOXP3^+^ T suppressor cells [16] (ii) IL-10-secreting CD8^+^ CD28^−^ FOXP3^−^ T suppressor cells [17], (iii) CD8^+^ CD57^+^ T suppressor cells [18].

CD8^+^CD28^–^ T cells, prevent T cell activation through direct cell contact either with effector T cells or with APCs [19]. This regulatory cell population expresses FOXP3 mRNA, uses a limited TCR repertoire and recognizes peptides presented within class I MHC molecules [19, 20]. As reported by Korecka-Polak et al. this cellular subset does not express FOXP3 protein [21].

CD8^+^CD28^−^ T cells inhibit the activation of CD4^+^ T cells causing a reduced expression of several co-stimulatory molecules, including CD40, CD80, and CD86 and the up-regulation of the inhibitory receptors ILT3 and ILT4 on APCs, rendering them tolerogenic [20, 22]. The interaction between DCs and CD8^+^CD28^–^ T cells primes a cascade of events, which result in effector T cell unresponsiveness to alloantigens presented via both direct and indirect pathways and subsequent graft adaptation [20, 22].

Differentially, CD8^+^ CD28^−^ FOXP3^−^ T suppressor cells are non-antigen-specific and they do not express CD56 and CD127 molecules. Additionally, they are anergic and this condition cannot be overcome under mitogens [23]. In vitro CD8^+^ CD28^−^ FOXP3^−^ T suppressor cells are generated from CD8^+^ CD28^−^ T cells after stimulation with IL-2 and IL-10 without the need for TCR stimulation [24]. They inhibit the APC activity of DCs, T-cell proliferation and cytotoxicity of cytotoxic T lymphocyte through the secretion of IL-10 [17, 23, 25].

CD8^+^ CD57^+^ T suppressor cells are commonly found in individuals with chronic immune activation and in clinical conditions characterized by functional immune deficiency, including human immunodeficiency virus (HIV) and cytomegalovirus (CMV) infection [26]. These cells are characterized by cytotoxic potential due to the high levels of granzymes and perforin [27], and produce the immunomodulatory cytokines IFN-ɣ and TNF-α [28].

Recently, a novel subset of regulatory CD4^−^CD8^−^ double-negative (DN) T cells was described. DN T cells have the same features of terminally differentiated effector memory T cells reexpressing CD45RA^+^ (T_EMRA_), but are CD27^+^CD28^+^ and do not express the transcription factor T-bet which is highly expressed in T_EMRA_ cells [29].

DN T cells modulate immune responses mediated by effector CD4^+^ and CD8^+^ T cells and prevent allograft rejection [30]. These cells may exert their immunosuppressive effects through several mechanisms. They are able to kill T cells in an antigen-specific fashion through the activation of the CD95–CD95L pathway, they can induce DC apoptosis, they can acquire alloantigens from DCs by trogocytosis and they cause a significant reduction in CD80 and CD86 expression on DCs surface. Particularly, this process requires CTLA4 expression by DN Tregs, since CTLA4^−^ DN T were totally unable to downregulate the costimulatory molecules on DCs surface [29].

It has been demonstrated that tolerogenic DCs induced IFN-*γ* expression in DN T cells leading to their accumulation in the spleens of operationally tolerant rats. Noteworthy, IFN-*γ* blockade in this setting resulted in allograft rejection [31].

Interleukin-7, that plays an important role in the homeostasis of the T cell compartment, can decrease the suppressive activity of DN T cells activating the Akt/mTOR pathway in human DN T cells. Interestingly, selective inhibition of Akt/mTOR signaling has an opposite effect to IL-7 and restores the functionality of DN T cells [32].

Tregs can develop via two different pathways. Naturally occurring or Thymus-derived Tregs, known as CD4^+^CD25^+^FoxP3^+^ Tregs, are selected in the thymus and exert their actions in the periphery usually to suppress responses to self-antigens. On the other hand, naive T cells meeting the antigen in the periphery in a tolerogenic microenvironment may differentiate into inducible Tregs (iTregs). The induction of Foxp3 expression, essential for maintenance of tolerogenic characteristics of Treg, in CD4^+^CD25^−^ T cells is induced by IL-2 and TGF-β [33–38], together with a suboptimal stimulation of TCR.

In particular in the gut-associated lymphoid tissues (GALT) functionally specialized intestinal DC that express the integrin CD103 can induce gut-homing receptors on naïve CD4^+^ T cells through a mechanism depending on TGF-β and retinoic acid [35, 39–41].

The best studied subset of iTregs is the Tr1 cells which, in contrast to FoxP3^+^Tregs, lack FoxP3 expression and any lineage-specification transcription factor. They modulate T cell functions secreting particularly high levels of IL-10 [42]. For this feature, Tr1 cells represent one of the main T-cell mediators of cytokine-dependent immune regulation in both mice and humans and, accordingly, Foxp3^+^Treg and Tr1 cells are considered two distinct subsets of Treg cells [42].

Several in vivo and in vitro observations suggest an impact of rapamycin on both Tregs’ populations. In murine models rapamycin, but not CNI, induces the proliferation and the regulatory effects of naturally occurring Tregs [43]. Battaglia et al. [44] reported that in vitro activation of CD4^+^ T cells, obtained by healthy subjects or type 1 diabetic patients, in the presence of an mTOR inhibitor induces the expansion of CD4^+^CD25^+^FoxP3-Tregs, which, in turn, inhibit syngeneic and allogeneic CD4^+^ and CD8^+^ T cell proliferation. Interestingly, they demonstrated that rapamycin, unlike CNIs, inhibiting the proliferation of effector T cells, spares and induces the growth of circulating Tregs and these cells show the ability to be expanded preserving their suppressive activity. In addition, several studies suggested that rapamycin might also induce the development of Tregs in mixed lymphocyte cultures [45]. Interestingly, in this setting, Tregs were not generated through the expansion of naturally occurring regulatory T cells, but by the induction of a regulatory phenotype in conventional CD4^+^ T cells.

Moreover rapamycin resulted in enhanced Foxp3 expression at high dose of anti-CD3 and anti-CD28 stimulation. This effect is dependent on endogenous TGF-β since significantly reduced frequencies of Foxp3-expressing CD4^+^ T cells were detected in the presence of anti-TGF-β antibody [46].

Therefore, mTOR inhibition can both expand naturally occurring Tregs and induce adaptive Tregs from conventional CD4^+^ T cells. In addition, it has been recently demonstrated that rapamycin can also increase Tregs donor-specific suppressive ability [47]. It should be considered that the inhibitory effects of rapamycin on cytokine expression and T-cell differentiation might be cell specific, thus favoring Tregs expansion over of effector T cells differentiation. Thus, it is conceivable that CD25 signaling through mTOR is absolutely needed for effector T cells differentiation, whereas Tregs may use an “escape” signaling pathway. This hypothesis is supported by the observation that in the presence of IL-2, rapamycin alone or combined with stimulation delivered by TCR and CD28 can foster the selective proliferation of naturally occurring Tregs [48]. IL-2 administration to pediatric patients with sarcoma during immune reconstitution significantly increased peripheral Tregs number compared to patients not receiving the cytokine therapy [49]. Similarly, in vitro observations suggest that cyclosporine A, inhibiting NFAT translocation into the nucleus, suppress FOXP3 promoter activity and subsequently its gene and protein expression in T cells [50]. In accordance, Pascual et al. [51] demonstrated that after withdrawal of CNIs in renal transplant patients, a significantly increase of Tregs in peripheral blood could be observed in comparison with patients remaining with CNI, indirectly suggesting the previously described limitation of CNIs to favor the expansion of Tregs.

The mTOR inhibitors have provided a new powerful immunosuppressants potentially able to replace CNIs in kidney transplantation. The exact unique immunosuppressive profile of Sirolimus in humans was investigated in vivo by Brouard et al. [52] by employing phenotypic analysis on peripheral blood mononuclear cells (PBMCs) harvested from stable kidney recipients under immunosuppressive therapy with Sirolimus or CNIs, they demonstrated that Sirolimus-treated recipients have a larger population of CD4^+^CD25^+^Foxp3^+^ Treg cells than those under CNIs [52]. In the clinical setting, the main therapeutic approach for reaching a tolerogenic state has been mainly based on recipient T-cell depletion and then, the use of minimal post-transplant immunosuppression based on mTOR inhibitors [53, 54] as maintenance immunosuppressive drugs. In these studies, authors showed a significant increase in the total number of circulating Tregs until 2 years after transplantation, and most interestingly, these Tregs were responsible of the anti-donor hypo-responsiveness achieved in some patients as demonstrated by their anti-donor suppressive activity ex vivo. Also, patients displaying donor-specific hypo-responsiveness showed a significantly high proportion of Tregs within cellular graft infiltrates.

### DCs and mTOR inhibition

The DCs have attracted much interest in the medical community during the last years due to their strategic role in immune response linking innate and acquired immune responses and in the induction of tolerance to self and non-self antigens. Their capacity to regulate T-cell responses reflects the ability to provide critical instructive signals mediated by several specific co-stimulatory molecules such as B7, tumor necrosis factor family members (OX40, CD40, CD70), and specific cytokines. Adequate activation of these receptors results in DCs “maturation,” enhancing their ability to activate effector CD4^+^ and CD8^+^ T cells. On the contrary, immature and “semi-immature” DCs populations, which lack adequate T-cell stimulatory ability, can suppress peripheral Ag-specific immune responses through the induction of T-cell anergy and deletion. In alloimmunity, the ability of both immature donor and recipient-derived DCs to suppress alloantigen-specific immune responses and prolong graft survival has been demonstrated in animal models. Both “immature” and “mature” DCs has been shown to be able to initiate the expansion of Tregs. Therefore, T-cell responses could be viewed as the culmination of a DCs-driven expansion and induction of effector functions of both regulatory and non-regulatory T cells. In fact, there are several studies showing the capacity of DCs to expand/induce Tregs [55–57]. Tissue-resident immature DC presenting low numbers of self peptide-MHC complexes coupled with limited co-stimulatory molecules expression can convert conventional naïve T cells into Treg probably as a consequence of their presentation of an antigen to T cells without concomitant costimulation of cytokines. Nevertheless mature DC may retain their tolerogenic function depending upon exposure to different stimuli. Activation of immature DC in the presence of IL-10, for instance, limits up-regulation of co-stimulatory molecules, decreases secretion of proinflammatory cytokines, and increases IL-10 production [58, 59] enhancing Treg differentiation [60, 61]. Likewise immature DCs exposed to TNFα or IFNɣ although acquire high levels of MHC and costimulatory molecules, induce preferentially Treg differentiation [60, 61].

Within recent years, a growing number of publications have demonstrated that several clinically established immunosuppressive drugs target not only the effector cells but modulate also key functions of DCs [56]. Knowledge about the specific pharmacological effects of immunosuppressive agents on DCs does not only provide novel insight into the biological mode of action but additionally allows the development of experimental and clinical protocols that promote the generation of tolerogenic antigen presenting cells. One key prerequisite, however, for DCs to exert immunoregulatory functions is the migration into lymphoid tissues, where they can interact intimately with lymphocytes [57]. Sordi et al. showed in vitro and in vivo data revealing that the immunosuppressive macrolide rapamycin increases surface expression of the chemokine receptor CCR7 on human and mouse derived DCs and consequently promotes DCs migration into lymphoid tissue [62]. In contrast, the authors did not find significant biological effects of CNIs cyclosporine and FK506 on DCs chemokine receptor expression and migration. Rapamycin was the first clinically relevant immunosuppressant to be shown to inhibit antigen uptake by DCs in vitro and in vivo [63]. The study by Sordi et al. [62] reveals unique immunomodulatory effects of this drug on the migratory behavior of DCs. The authors employed different animal models in order to confirm their in vitro observations of CCR7 up-regulation by rapamycin-exposed human monocyte-derived DCs. Moreover, it should be noted, that the in vitro experiments have been performed with clinically relevant concentrations of rapamycin (1–10 ng/ml). The in vivo migration experiments with murine bone marrow derived DCs exposed ex vivo to rapamycin demonstrated increased numbers of DCs in the regional lymph nodes after 24 h when compared to controls. These observations were further confirmed in a fluorescein-isothiocyanate skin-painting model with mice receiving a single dose of rapamycin orally. The in vitro experiments show that competition for rapamycin’s intracellular receptor FKBP12 with a molar excess of FK506 can prevent the CCR7 up-regulation induced by rapamycin. Thus, CCR7 up-regulation is likely to be a drug-specific effect related to the interaction of rapamycin with its intracellular receptor. The authors suggested that inhibition of endogenous IL-10 was at least partially responsible for the observed effects, since anti- IL-10 mAbs impaired rapamycin’s effect on CCR7 expression [62].

Taken together, these findings demonstrate for the first time that a potent immunosuppressive agent can promote the migration of professional antigen presenting cells into lymphoid tissue. This may sound contradictory if we see DCs only as potent stimulators of adaptive immune responses [62]. In fact, it makes a lot of sense, since an increasing number of studies have demonstrated that so called semi mature DCs transport continuously self-antigens into the T cell areas of lymphoid tissue and are important inducers of Tregs [64]. Thus, rapamycin’s pro-migratory effect on DCs indicates that this agent may exhibit immunoregulatory potential. With respect to this conclusion it is interesting to note, that the Pittsburgh group [65] reported that rapamycin-treated, alloantigen-pulsed DCs induce antigen-specific regulation and prolong experimental heart allograft survival. Taner et al. demonstrated that rapamycin-exposed DCs could be loaded with donor cell lysates and that infusion of these DCs prior to experimental heart transplantation prolonged fully MHC mismatched murine heart allograft survival [65]. Interestingly, this effect was enhanced by repeated infusion of the cells. Taking into account all these findings rapamycin not only keeps DCs moving, but reveals novel aspects of pharmacological immunosuppression. In fact, these drugs inhibit DCs maturation and thus, T-cell stimulatory capacity both in vitro and in vivo at clinically relevant levels.

**Fig. 1:**
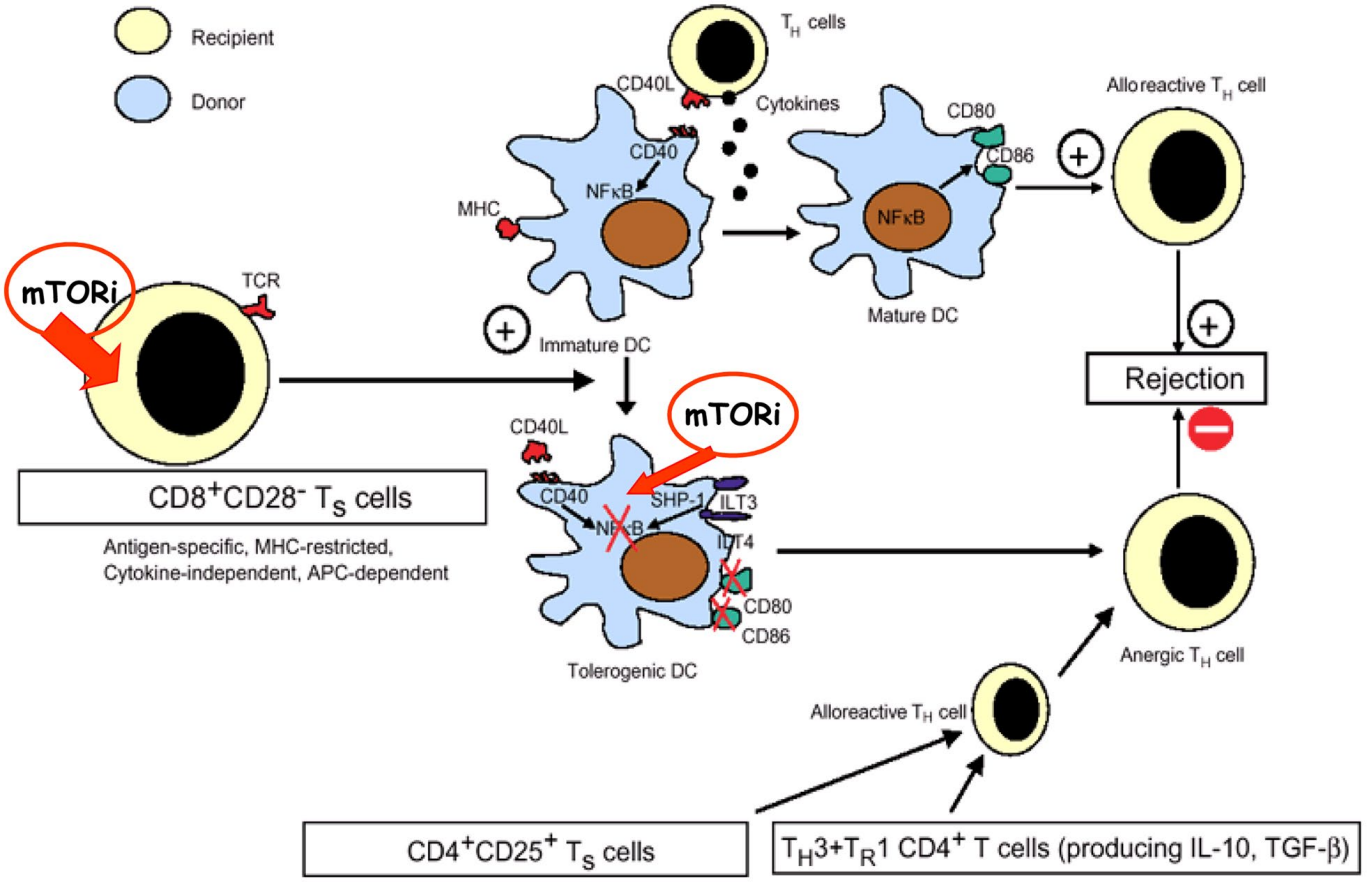
A potential mTOR inhibithor-modulated immune mechanism. The *figure* summarized the possible tolerogenic effect induced by mTOR inhibition through a specific sequence of events characterized by an increase in circulating C8^+^C28^−^ Ts followed by a significant increase of ILT3 and ILT4 expression on circulating DCs due to a strong reduction of CD40 expression Adapted from Mark B. Feinberg & Guido Silvestri: TS cells and immune tolerance induction: a regulatory renaissance? Nature Immunology 2002; 3: 215–217

Recently, we focused our attention on the inhibitory receptors ILT3 (Ig-like transcript 3) and ILT4 (Ig-like transcript 4) that are crucial to the tolerogenic phenotype acquired by professional and non-professional APC, such as dendritic and endothelial cells, respectively. ILT3 and ILT4 belong to the Ig superfamily and are selectively expressed by professional and nonprofessional antigen-presenting cells, including monocytes, macrophages, DCs, and endothelial cells [66, 67]. Similar to other members of the Ig superfamily, ILT3 and ILT4 present extracellular Ig-like domains and a long cytoplasmic tail containing an ITIM-like motif, which recruits inhibitory phosphatases and transduces negative signals. IL-10 and/or interferon-alfa induce ILT3 and ILT4 expression in DCs and endothelial cells and promote their ability to inhibit the proliferation of allogeneic T cells [22]. We demonstrated that rapamycin induces an increase in circulating plasmacytoid DCs with a significant up-regulation of ILT3 and ILT4 and a reduction of CD40 expression on their cell surface. These changes were associated with an increase in circulating CD8^+^CD28^−^T cells and CD4^+^CD25^+^Foxp3^+^CTLA4^+^ Tregs and with a significant switch from a Th1 to a Th2 bias in circulating and graft-infiltrating T cells [68]. Besides a significant change in DCs phenotype, it is conceivable that rapamycin may also influence the main DCs functions. Moreover, has been demonstrated that rapamycin attenuates the hypoxic immune-inflammatory response through inhibition of the Hypoxia-inducible factor 1α (HIF-1α) pathway thus, abrogating effector alloimmune responses [69]. In an interesting report [70], it has been shown that DCs generated in the presence of rapamycin are poor allostimulators and resistant to maturation after CD40 ligation. Outstandingly, although their T-cell allostimulatory ability was markedly impaired, rapamycin-DCs skew the balance of FoxP3-Tregs relative to T effectors, by maintaining the ability to stimulate Treg similar to control DCs. Moreover, when a single infusion of recipient-derived, alloantigen-pulsed rapamycin-treated-DCs, combined with a short course of minimally effective rapamycin was performed, indefinite organ graft survival could be observed and it was associated with graft infiltration by Treg and the absence of transplant vasculopathy [70]. Therefore, it seems that mTOR inhibitors may actively play a role in DCs to allow Treg activation while minimizing effector T-cell activation, thus favoring a tolerogenic state (Fig. 1).

### Myeloid derived suppressor cells (MDSC) and mTOR inhibition

MDSC are a group of myeloid cells comprised of precursors of macrophages, dendritic cells, granulocytes and myeloid cells at early stage of differentiation with immune suppressive activity [71, 72].

MDSC can be divided in two groups, monocytic and granulocytic, based on phenotypic and functional features [73]. Both groups express high levels of CD11b, CD33 and MHC class I, and low or absent expression of MHC class II molecules [74]. Conversely, they can be easily differentiated according to the expression of Ly6C (high in monocytic and medium in granulocytic MDSC) and CD14 (present only in monocytic MDSC).

These cells regulate immune response in cancer [71, 75, 76], bacterial/parasitic infections, acute or chronic inflammation, traumatic stress, autoimmune disease and transplantation [77–81] by an intricate biological machinery. In particular, MDSCs, expressing high levels of both arginase 1 and inducible nitric oxide synthase (iNOS), may determine a significant depletion of l-Arginine in the microenvironment with a consequent decrease of the T cell proliferative capacity [82]. Moreover, NO released by iNOS suppresses T-cell function by the inhibition of JAK3, STAT5 and MHC class II [83–85]. Then, because of the depletion of arginine, iNOS may preferentially produce superoxide that, together with NO, generate peroxynitrite resulting in a nitration of TCR and impairment of T cell peptide/MHC interaction [86]. Other mechanisms proposed involve heme oxygenase-1 (HO-1), TGF-β and indoleamine 2,3-dioxygenase (IDO) [87–89].

The influence of mTOR inhibition on MDSCs proliferation/function has been recently elucidated by Wu et al. [90] in alloskin-grafted and tumour-bearing mouse models. In both models, mTOR inhibition (by rapamycin or mTOR KO) decreased percentages and number of monocytic MDSCs and reduced immunosuppressive activity. The latter effect seemed to be due to the mTOR-I-related inhibition of iNOS and arginase activities. Contrarily, none of these effects was seen in granulocytic MDSCs.

Rapamycin, then, was also able to decrease monocytic MDSC differentiation from myeloid progenitors by blocking the glycolysis, an essential pathway involved in MDSCs differentiation.

## Summary

### mTOR inhibitors and tolerance induction

The exact biological mechanisms underlying mTOR inhibitors tolerogenic effects are still largely unclear. Indeed, these immunosuppressive agents may modulate an array of signaling pathways. The potential molecular mechanisms have been mostly studied in animal models, although some relevant insights have been also reported in humans. The presence of T-cell immunoregulation has been defined to be the hallmark of peripheral allograft tolerance. Two main immunologic cellular populations have been described to play a key role in this setting: CD4-Tregs and DCs. In fact, several reports have shown that the presence of activated CD4-Tregs play a critical role in controlling undesired immune responses to self and non-self-antigens, to prevent autoimmune diseases and to achieve peripheral allograft tolerance [91, 92]. Likewise, although professional antigen-presenting DCs are known to be key initiators of effector alloimmune responses after transplantation, they have proved to be able to modulate alloimmune responses inducing antigen specific Tregs or directly suppressing peripheral antigen-specific alloimmune responses through the induction of T-cell anergy and deletion [91, 92]. Outstandingly, mTOR inhibitors have been shown to modulate the activities of these two particular cellular populations.

## Conclusions

Rapamycin, a macrolide antibiotic produced by *Streptomyces hygroscopicus*, is an effective immunosuppressive drug used to prevent allograft rejection [5]. Similarly to the immunosuppressants FK506 and cyclosporine A (CsA), rapamycin exerts its effects binding to cyclophilin. However, unlike FK506 and CsA, rapamycin does not inhibit T-cell receptor (TCR)–induced calcineurin activity. Rather, the rapamycin-FKBP12 complex inhibits the serine/threonine protein kinase mTOR, the activation of which is required for protein synthesis and cell cycle progression. Rapamycin blocks signaling in response to cytokines/growth factors, whereas FK506 and CsA exert their inhibitory effects blocking TCR-induced activation [5]. Consistent with this mechanism of action, it has been shown that rapamycin blocks T-cell-cycle progression from G1 to S phase after activation, promotes TCR-induced T-cell anergy even in the presence of co-stimulation, and allows induction of operational tolerance. Additionally, it has been shown that mTOR takes part in several signalling pathways potentially involved in oncogenesis [93].

The great success achieved in organ transplantation in the 1990s drove investigators to look for refinements in immunosuppression. This success was based on the excellent acute rejection prophylaxis provided by the CNIs. However, long-term allograft survival has been shown to be limited in the last decade, without the improvement that would have been expected [94]. In fact, among the main reasons, the chronic nephrotoxic effect of CNIs, leading to allograft scarring, induce the transplant community to seek for new non-nephrotoxic immunosuppressive drugs such as mTOR inhibitors. Nevertheless, after 10 years of clinical experience mTOR inhibitors are still far to be positioned as a first-line immunosuppression in organ transplantation. Unexpectedly, CNIs are still considered as the cornerstone of immunosuppression. Indeed, there are several reasons for justifying this failure of mTOR inhibitors, including an increase in surgical and wound-healing complications, deferred recovery from delayed graft function in renal transplantation, the limits in anti-rejection effects in the presence of effector memory T cells, the association with post-transplant proteinuria and new onset diabetes after transplant (NODAT). However, there is increasing body of evidence supporting that more than playing an immunosuppressive role, mTOR inhibitors display several, relevant biological properties including anti-neoplastic activity, cardiovascular protective actions, and specific immunomodulatory effects that might facilitate the achievement of operational allograft tolerance. Importantly, transplant tolerance is expected to reduce rejection while minimizing immunosuppressive side effects, potentially leading to a significant improvement of outcome in the long term. In this setting, mTOR inhibitors through different biological mechanisms have been shown to play a key role favoring this immune privilege state both in animal models and in humans.
